# A Sensing and Monitoring System for Hydrodynamic Flow Based on Imaging and Ultrasound

**DOI:** 10.3390/s19061347

**Published:** 2019-03-18

**Authors:** Aimé Lay-Ekuakille, Vito Telesca, Giuseppina Anna Giorgio

**Affiliations:** 1 Department of Innovation Engineering, University of Salento, 73100 Lecce, Italy; 2 School of Engineering, University of Basilicata, 85100 Potenza, Italy; vito.telesca@unibas.it (V.T.); giuseppina.giorgio@unibas.it (G.A.G.)

**Keywords:** open channel and conduit monitoring, hydrodynamic monitoring, water flow monitoring, structural health monitoring, sensors and sensing systems, ultrasound sensors, particle image velocimetry, built environment

## Abstract

A built environment, that also includes infrastructures, needs to be taken under control to prevent unexpected modifications, otherwise it could react as a loose cannon. Sensing techniques and technologies can come to the rescue of built environments thanks to their capabilities to monitor appropriately. This article illustrates findings related to monitoring a channel hydrodynamic behavior by means of sensors based on imaging and ultrasound. The ultrasound approach is used here to monitor the height of the water with respect to a maximum limit. Imaging treatment is here proposed to understand the flow velocity under the area to be considered. Since these areas can be covered by trash, an enhanced version of the particle image velocimetry technique has been implemented, allowing the discrimination of trash from water flow. Even in the presence of the total area occupied by trash, it is able to detect the velocity of particles underneath. Rainfall and hydraulic levels have been included and processed to strengthen the study.

## 1. Introduction 

The understanding of the hydrodynamic behavior of water channels and rivers allows crucial information to be discovered about a possible dangerous area. This information is very important in risk management of the public authorities and to inform the public, regional, environmental, and protection agencies, and to raise alarms for people [1,2].

To predict urban flooding, estimating water levels is pivotal. A more effective and economically viable method for avoiding the harmful effects of hydrodynamic variations is to monitor the evolution of water channels over time and to implement the remediation works required [3].

Therefore, hydrodynamic monitoring must be assessed, also and above all, in the light of recent local climate change. Indeed, future climate projections suggest a potential increase in mean air temperature and precipitation [4]. The change is expected to alter the watershed characteristics, affecting the distribution of flows (velocity and water level) and suspended sediment concentration, and transport within the fluvial system [5].

River flood monitoring and control require measurement and notification of the water level, velocity, and meteorological data (precipitation). Aside from using historical data and specific domain knowledge to deliver more accurate flood forecasts, the river flood forecasting is based on space and ground-observed data received from satellites and terrestrial (meteorological and climatological) stations. These data may be represented as images, terrain information, and environmental information (i.e., drainage network, rainfall, and hydrology data) [6].

Understanding hydrodynamic flow in open channels and conduits is crucial for hydrology and hydraulic engineering practice [7]. Flow gauging relies on local water level and surface-flow velocity measurements through traditional (water level meters in gauging stations or portable flow meters) and alternative methods (ultrasonic meters, microwave sensors, and radar). Traditional methods need sampling and are intrusive, whilst innovative approaches are relatively expensive and typically (such as satellite techniques) feasible only for large river water depths [8,9]. The existing tools that rely on the historical record may be insufficient to identify these variations in flow hydrodynamic behavior [10]. Therefore, in the last few years, novel hydro-environmental studies based on optical and imaging methods have been proposed to overcome some of these inconveniences [8,11,12,13,14].

Imaging techniques use flow images as data and apply similarity and pattern recognition algorithms to obtain quantitative information on the flow [8].

Moreover it is also possible to use ultrasonic sensors to develop an effective monitoring system and to ensure adequate transmission rates and to prevent data loss [15,16,17,18]. Level measurement of the river/channel by ultrasound remains one the most important techniques because it is independent from temperature. It can be used in connection with flow measurement to calculate the time that ranges from one level to another. This time is entirely dependent upon flow velocity.

**Figure 1:**
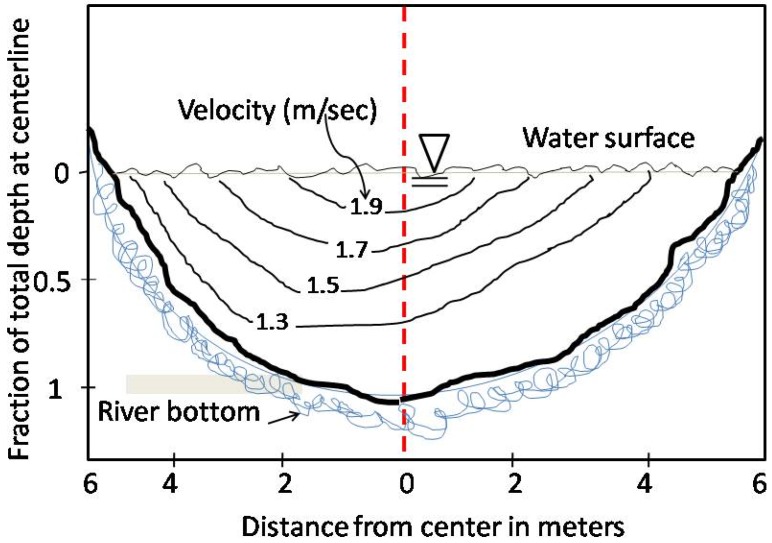
Cross section of a water channel/river, illustration of depth fraction as function of section length.

**Figure 2:**
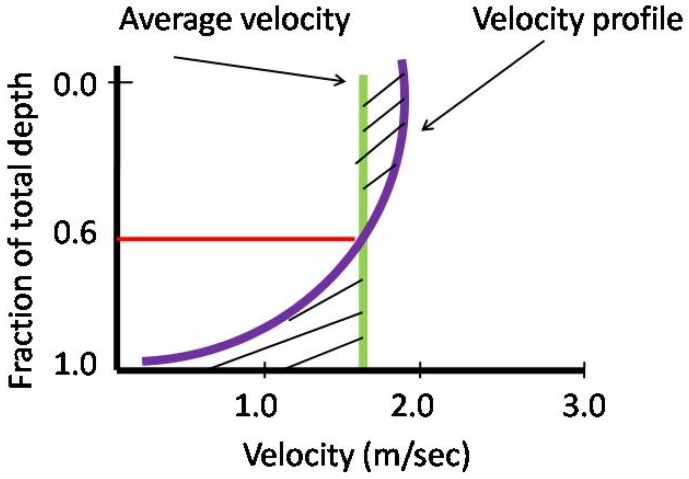
Fractional depth as function of velocity in a channel and velocity profile.

There are diverse techniques for monitoring river/channel flow given its structural and dynamic conditions. The channel is per se an infrastructure similar to a pipeline to be monitored [19,20]. First of all, let us consider a classical configuration of a channel (equivalent to a river) as represented in Figure 1 in terms of cross–section, and velocity profile as in Figure 2. Different types of velocities are involved for this type of infrastructure: the velocity at the surface (*v_s_*) and average velocity (*v_avg_*) at a given section, as illustrated in Figure 2.

Therefore we can define the flow in terms of the average velocity (*v_avg_*) and average area (*A_avg_*) at a given section.

Going back to techniques, the following ones, based on stream gauging, are mostly used:

- Mechanical meters [21]: here, velocity is measured by locating a meter in a stream and counting the number of revolutions in a measured quantity if time. With the same technique, velocity of profile, and velocity area method are carried out. Velocity area method [22] is the calculation of discharge which is the product of water velocity times the area of water in cross section.
- Acoustic meters [23]: these are based on measured times for acoustic pulses to traverse a river section along an oblique path in both directions. The mean river velocity is related to the difference in the two timings, and the flow is then assessed using the river’s cross-sectional area.
- Volumetric flow rate of water [24]: this is generally evaluated from the measured water-surface elevation (stage) by means of an empirical fit to measurements of stage and concurrent discharge.
- Flumes [25]: generally portable, used for low flow measurements in channels.

An important issue is measurement processing either raw signals or numerical data. For this approach, advanced methods are used. Measurements of water levels in the main channels of rivers, tributaries, and lakes are necessary for understanding flooding hazards, sediment transport, and nutrient exchange [26]. This paper shows the outcomes of monitoring a Tara channel (Apulia, Southern Italy) hydrodynamic behavior by means of sensors based on imaging and ultrasound. In particular, in order to understand the flow and height monitoring by ultrasound, the imaging treatment is used. Moreover, the height monitoring by ultrasound is described.

## 2. Proposed Enhanced Imaging Technique for Water Flow Monitoring

Since the instrumentation mounted on the experimental channels is, notably, an acoustic system for the water level and a camera for checking the presence of trash and debris on the surface of the channel, the proposed technique of flow and velocity detection is based on particle image velocimetry (PIV).

The PIV method (particle image velocity) is an imaging technique designed to measure the velocity field in a flow. To obtain a good measurement resolution, a high concentration of tracer is used.

For recording images or a sequence of PIV, the flow is inoculated with micro particles as indicators. In order to have particles entrained by the global motion at non-zero acceleration, it must be ensured that the velocity is as close as possible to that of the fluid. The choice will also depend on the particle’s ability to redistribute light from the laser plane to the shooting plane. Here, given the low flow of the channel, it is difficult to apply PIV. However, PIV is here enhanced to reduce noise in the processing so that particles are clearly discriminated.

The basic technique consists in cutting out the two images from the same pair of equivalent interrogation windows and correlating them between two corresponding interrogation windows. The maximum position of the correlation map by in the center of the map gives the value of the most likely movement of the particles in the interrogation window [27].

**Figure 3:**
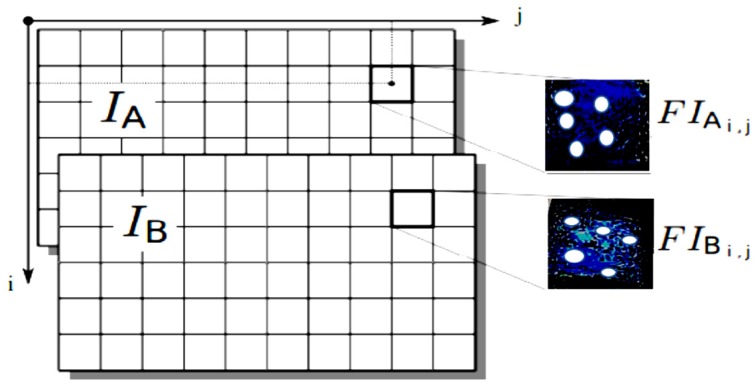
Splitting a pair of images into interrogation windows (extract from [29]).

**Figure 4:**
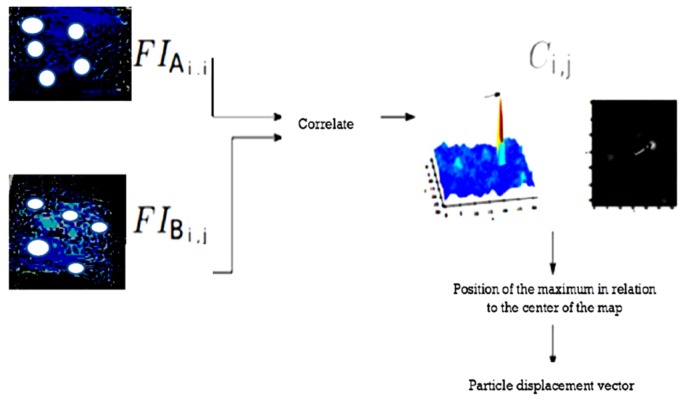
Calculation of the particle displacement in an interrogation window with pixel accuracy (extracted from [29]).

First of all, it is a matter of splitting the individual images into interrogation windows (Figure 3). The images *I_A_* and *I_B_* are respectively cut into *FI_A_* and *FI_B_*. Then, for each *FI_ij_*, the correlation between $FI_{A_{i,j}}$ and $FI_{B_{i,j}}$ gives a correlation map [28], as depicted in Figure 4.

In a continuous case, the correlation between two images $FI_{A_{i,j}}$ and $FI_{B_{i,j}}$ is as follows [29]:

(1) $$C_{i,i}(x,y)=\;\iint_{FI_{i,j}}FI_{A_{i,j}}(m,n)\times FI_{B_{i,j}}(m+x,n+y)dndm$$

In the direct case that interests us for digital images, if the images are sizes (2*m*,2*n*) the correlation is:

(2) $$C_{i,j}(x,y)=\;\sum_{m=-M}^{M}\sum_{n=-N}^{N}FI_{Ai,j}(m,n)\times FI_{B_{i,j}}(m+x,n+y)$$

For each position (x,y), we perform the point-by-point multiplication of $FI_{B_{i,j}}$ the translated image from (x,y). Then we add all the points of the obtained image. When the translation makes the two $FI_{i,j}$ overlap, then the maxima of each is multiplied together and the final sum is greater than all possible (x,y) couples. In general, this correlation is performed by a Fast Fourier Transformation (FFT). Indeed, the correlation between two images A and B is equivalent to a complex multiplication of the FFT of image B with the conjugate of the FFT of image A. We therefore carry out this multiplication, then we apply the Reverse Fast Fourier Transform (RFFT) to it, of which we take the real part. The correlation map $C_{i,j}$ for a pair of FI is then:

(3) $$C_{i,j}={ℜ}_{e}(TFRI(TFR(FI_{B_{i,j}})\times {\left(TFR(FI_{A_{i,j}})\right)}^{*}))$$

The position of the maximum being obtained in pixels, if we process the processing process at this step, gives an accuracy on the result to the pixel. Once we have obtained the displacements of the particles on the image in pixels, it remains to convert this to meters. This is achieved by a spatial calibration that associates a number of pixels on an image with a real distance. The speed, on the other hand, will be determined by taking into account of the delay between the two laser pulses.

**Figure 5:**
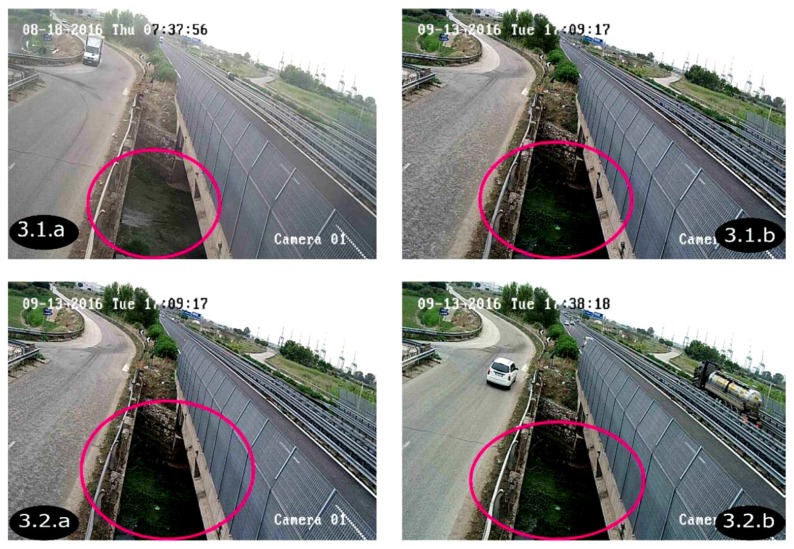
The images from the camera.

For each of the images of the experiment undertaken, pairs of images were recorded by each camera at an acquisition frequency around 1 Hz with an inter-frame time around 3.5 ms (milliseconds). For symmetry reasons, the analysis developed in this section is only performed on the images taken by camera 1 (Figure 5). These images are used to highlight the detection technique. Images labelled 3.1.a and 3.1.b are related to two different dates and times; and are used to verify diverse conditions. Whilst 3.2.a and 3.2.b are of the same day but different instances which are close together: an interval of 29 min. This choice is also important to detect subsequent particles for the algorithm.

**Figure 6:**
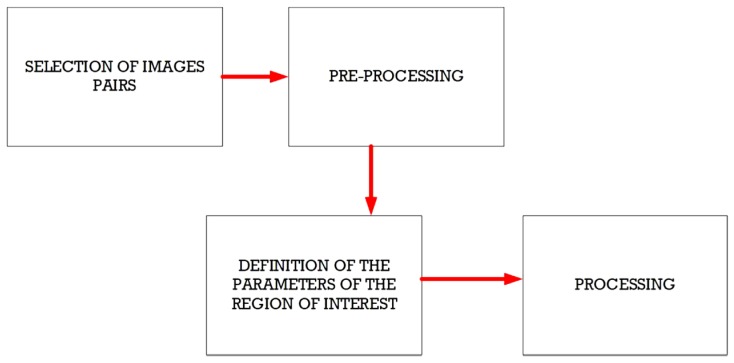
Steps required for PIV treatment.

An overall scheme of the proposed method is illustrated in Figure 6 where the region of interest is a clear issue for defining the target within the flow.

**Figure 7:**
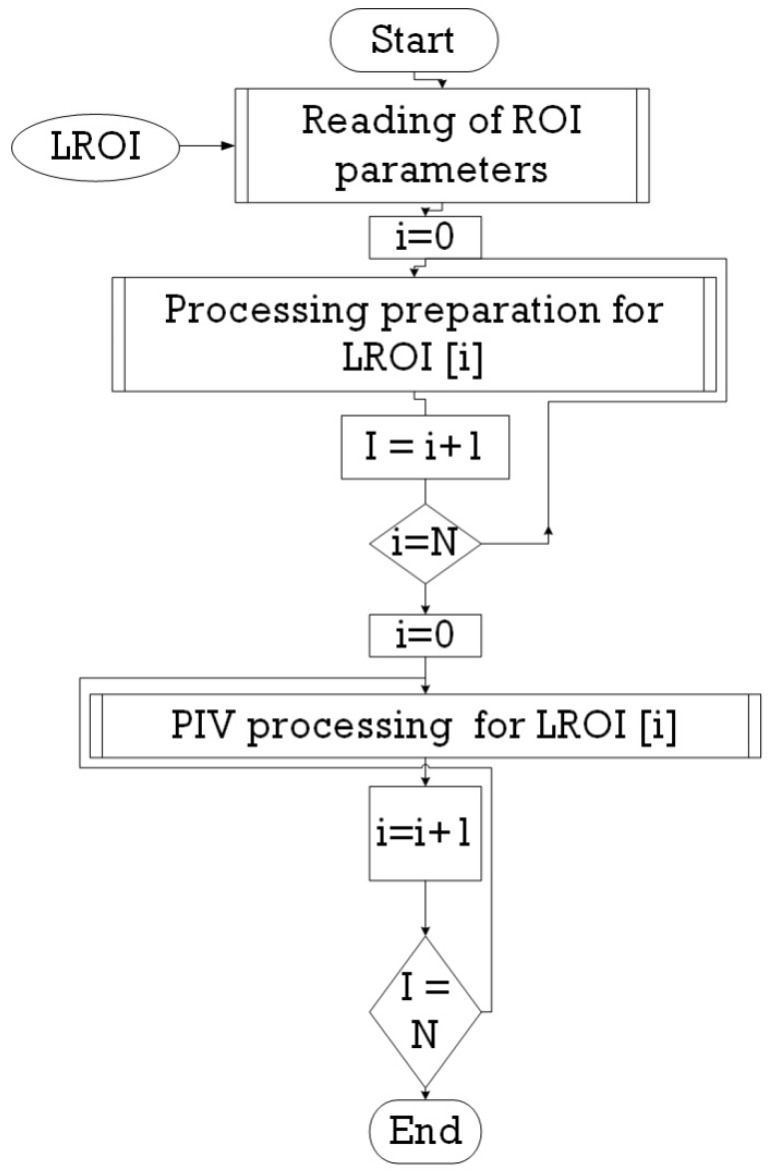
The general organization chart of the processing operation.

The organizational chart proposed by this work is as follows (Figure 7): at least two images or more are acquired by determining the region of interest (ROI). These regions are processed by splicing them in windows. The PIV algorithm is activated to find cross-correlation between two images (for example), as per Equation (2). The peak of such correlation is searched for, giving the displacement of the particle. The velocity vector corresponds to the peak position due to the displacement peak between the first and the second one.

## 3. Channel under Monitoring and Sensing Equipment

**Figure 8:**
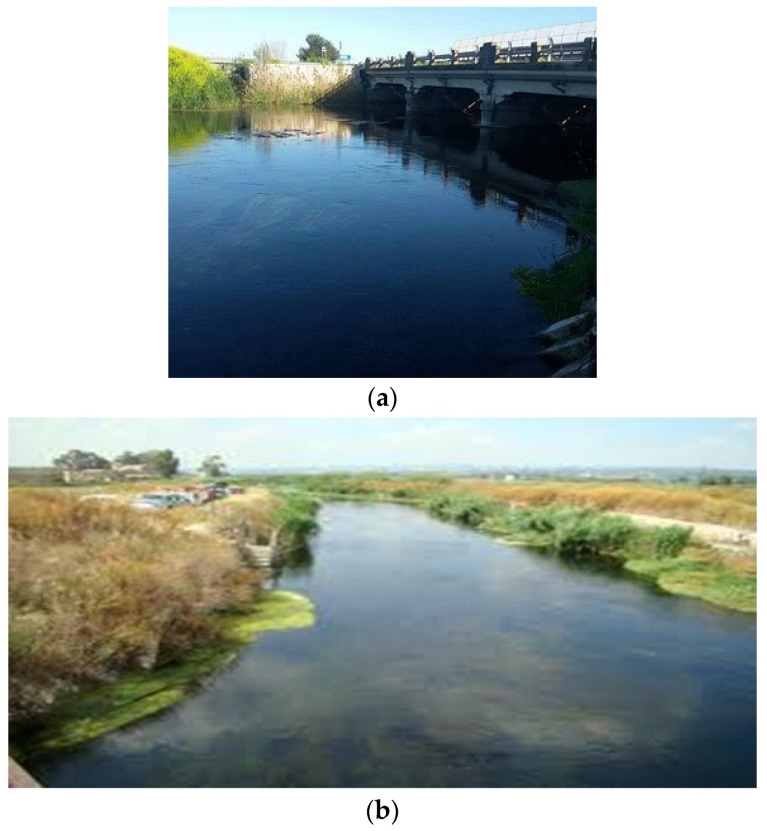
Downstream channel area which is opposite the monitoring station: (**a**) close to the bridge and (**b**) far away.

**Figure 9:**
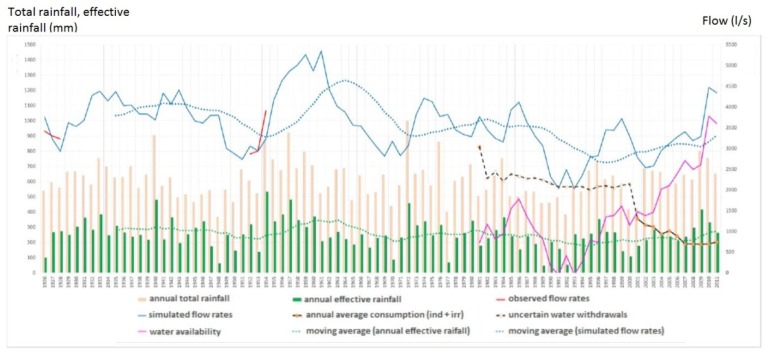
Hydrological balance with different terms related to flow rates, and rainfall: different hydraulic quantities of the Tara river. The fuchsia color indicates the so-called “water availability curve”.

**Figure 10:**
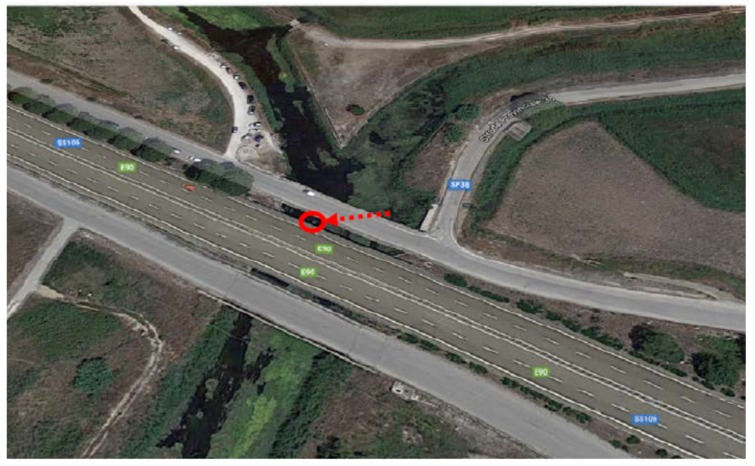
Location of the monitoring station on the road bridge, with a camera and ultrasound meter.

The channel under test and monitoring is called “Tara”, located in a western suburb of the city of Taranto (Italy). Its length is around 10 km. It was chosen because of recurrent floods that often devastate the neighboring areas where agricultural activities take place. The channel passes under three important bridges related to: a railway connecting the city to the southwest and northwest parts of Italy, the SS100 highway going west (Figure 8a), and juxtaposed to a local road going towards the maritime and industrial suburbs. According to Ancient Greek mythology (Magna Grecia), the channel, as a river, was used by Taras (*Τάρας*), son of Poseidon and Nympha, who came to be the founder of Taranto, around 2000 years BC. The Tara river discharges into the Jonian Sea. As it is clear to understand, monitoring the channel flow and water height is essential to protect vital infrastructures for daily activities impacting on the local and regional economy. The channel exhibits its hydraulic and hydrological features with periods of instabilities due to abundant and sudden rainfalls (Figure 9). Many human activities, especially agriculture and breeding, which heavily affect even surrounding areas, have been laid out in safety conditions. Therefore, a monitoring station was installed beside the highway bridge as located in Figure 10. The monitoring system includes an ultrasound level meter, a camera for displaying water at the bottom of the bridge, and a transmitting apparatus, allowing every pre-set range, to send information to a central computer and data storage system.

**Figure 11:**
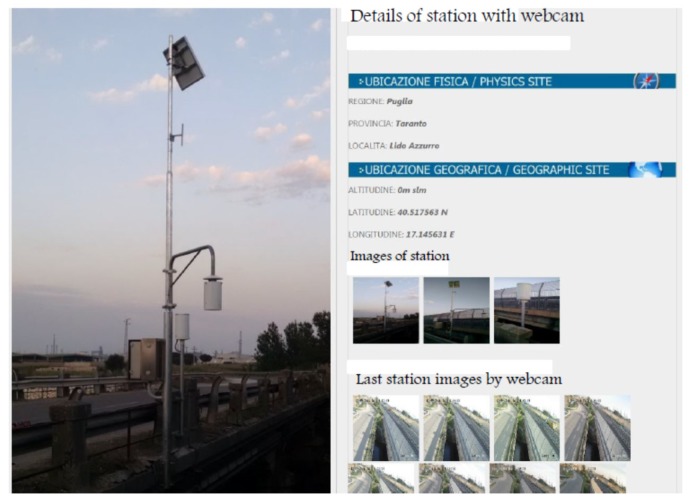
Monitoring station facility and its GPS coordinates.

The ultrasound level sensor, included in the station in Figure 11, using the ultrasonic measuring principle, records the time required for the sound wave to travel to the liquid surface and return. As the pulse propagation speed is highly influenced by air density and so by temperature, the measurement is automatically compensated for using the temperature value acquired locally by an integrated sensor.

The sensor is housed inside a glass reinforced Nylon enclosure with protection grade IP66/67; it is then installed inside a white aluminum shield to prevent direct solar irradiation and to provide at the same time correct ventilation. The sensor uses a non-contact method of detecting the level of water and it is characterized by the absence of moving mechanical parts. This way the ultrasonic system is virtually maintenance free. The transducer uses a two-wires connection for power supply and for the 4–20 mA analog output signal proportional to level measurement. The webcam transmits the photos within the same pack of level.

Once we have described the monitoring station and the channel hydraulic and hydrology, it is necessary to perform some metrics regarding the data acquired in the period of interest in 2016. The computation of these metrics isnecessary for subsequent validation and calibration.

## 4. Metrics for Testing and Validation

Nonparametric methods provide an alternative series of statistical methods that require no or very limited assumptions to be made about the data. There is a wide range of methods that can be used in different circumstances, but some of the more commonly used are ANOVA tests [26]. Nonparametric methods require no or very limited assumptions to be made about the format of the data, and they may therefore be preferable when the assumptions required for parametric methods are not valid. Nonparametric methods can be useful for dealing with unexpected, outlying observations that might be problematic with a parametric approach. These methods are intuitive and are simple to carry out by hand, for small samples at least [30].

The statistical analyses were all performed using XLSTAT tool. Selected nonparametric statistical methods were used in this study, including the sign test and the Wilcoxon signed rank test for paired data.

The chosen data set is related to the months of August and September; for the latitudes where the river is located, these two months represent a transitional period, that is, almost no rain in August before 20th and the beginning of occasional rainy days in September. This transitional period is also a source of instability, especially with strong and short rainfalls. It is the right period to test the increase of water level and velocity variations.

### 4.1. The Sign Test

The sign test is used to compare a single sample with some hypothesized value, and it is therefore of use in those situations in which the one-sample or paired t-test might traditionally be applied. This test is so called because it allocates a sign to each observation according to whether it lies above or below some hypothesized value, and does not take the magnitude of the observation into account. The sign test does not specify any underlined distribution and therefore it is a distribution-free statistic. If any observations are exactly equal to the hypothesized value, they are ignored and dropped from the sample. Exact *p* values for the sign test are based on the Binomial distribution. Note that the sign test merely explores the role of chance in explaining the relationship; it gives no direct estimate of the size of any effect. This lack of a straightforward effect estimate is an important drawback of nonparametric methods. A fixed significance level of 5% was selected for H0: μd = 0 [30,31].

### 4.2. The Wilcoxon Signed Rank Test

The sign test is intuitive and extremely simple to perform. However, one immediately obvious disadvantage is that it simply allocates a sign to each observation, according to whether it lies above or below some hypothesized value, and does not take the magnitude of the observation into account. An alternative that does account for the magnitude of the observations is the Wilcoxon signed rank test [30]. The Wilcoxon signed rank test may be applied in the same situations as the sign test, that is, for testing for (differences in) location in single populations or matched pairs, when the conditions for the corresponding *t*-tests are not met [31].

The Wilcoxon signed rank statistic subtracts the hypothesized median from the data. The absolute values of the resulting values, which are often called the centered data, are then ranked. Each rank is then given the sign of the centered data value. The test statistic, S+, is the sum of the ranks associated with positive centered data values. The test assumes that the data are independent and come from a continuous symmetric distribution [32]. Considering the choice images, two significant summer intervals were selected with a minor number of data missing and the same dimension of the series (Table 1).

**Figure 12:**
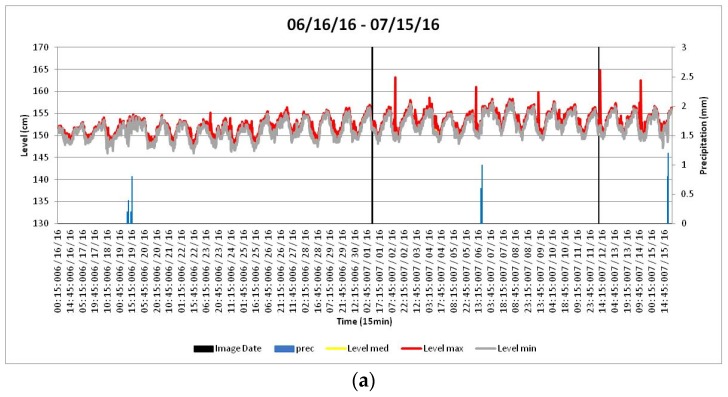

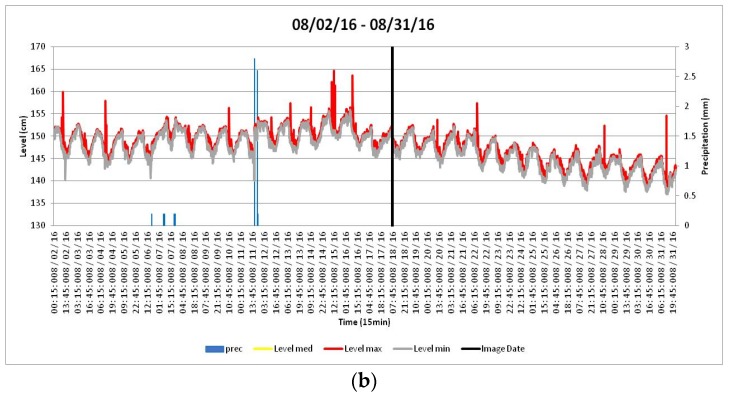
Different hydraulic levels.

For each period, description statistical analysis was performed. Table 2 reports the level values of average, maximum, minimum, standard deviation, and median. Moreover, the trends of minimum, maximum, and median levels and the precipitation are reported in Figure 12.

After the sign test, in the summer selection period, the Wilcoxon signed rank nonparametric test for all levels (maximum, medium, and minimum levels) was applied. *p* values less than 0.05 were considered statistically significant. For the hydrological level parameter, successive temporal paired-comparisons were chosen, namely, Level max_1 versus Level max_2, Level med_1 versus Level med_2, and Level min_1 versus Level min_2 (where the numbers 1 and 2 refer to the periods), were made with the Wilcoxon signed rank test. Chosen pairs are reported in Table 3.

The nonparametric statistical methods described were applied to analyze water level and quantify relationships between the two different periods.

## 5. Results

Given calculated metrics, as displayed in the subsequent subsection, are very important in order to correlate PIV method accuracy and sensitivity with respect to the detected velocity. Nonparametric tests have been chosen because of the non-homogeneity of the channel due to different reasons, notably, non-uniformity of its bed, presence of trash materials, anisotropy of the water surface, and solar impact on evapotranspiration. These factors affect image acquisition. This section is split into two subsections, namely, outputs from PIV techniques and results related to nonparametric method.

### 5.1. PIV Outputs

**Figure 13:**
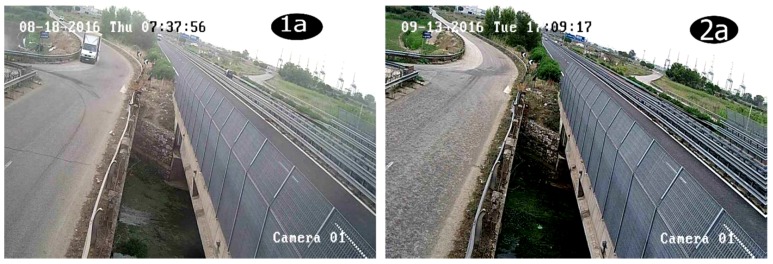
Original images (1a and 2a).

**Figure 14:**
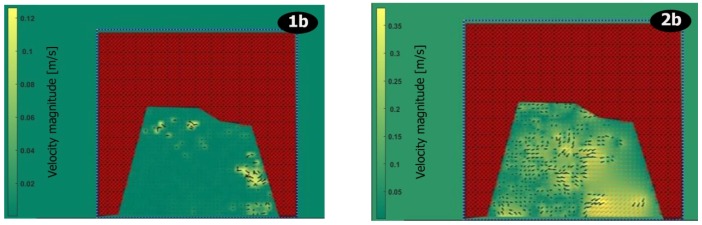
PIV treatment expressing the velocity field as velocity magnitude in m/s (1b and 2b).

In the first series of recordings, it is possible to make a clear observation of the velocity field as suggested by the PIV technique. The velocity field corresponds to a set of vectors moving away in order to yield to a resultant force from left to right under the bridge illustrated in the figures. That is, from the original images, captured by the webcam, photos 1a and 2a of Figure 13 are processed accordingly and bring to the velocity field of Figure 14 in which images 1b and 2b are associated with 1a and 2a respectively; water flow velocity is represented in the ordinates, ranging from 0.02 m/s up to 0.35 m/s. In photo 1a of Figure 14, the maximum speed is 0.12 m/s on 18 August 2016. That corresponds to a certain hydraulic level recalled in Figure 12; deep summer time. Conversely, in photo 2a of the same figure, the maximum speed is around 4 m/s on 13 September 2016; the beginning of the rainfalls, end of summer and approaching autumn. This reasoning is coherent to the period and the PIV has detected the right velocity field as further confirmed in the conclusions.

**Figure 15:**
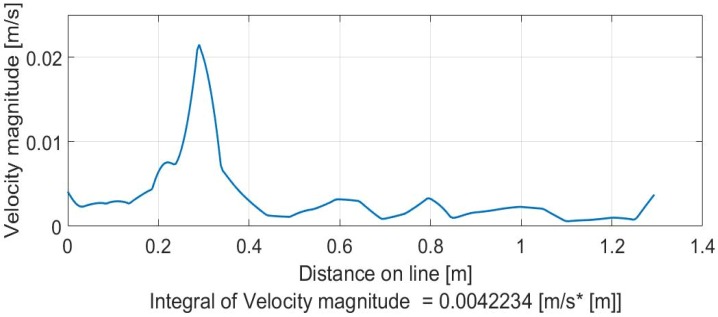

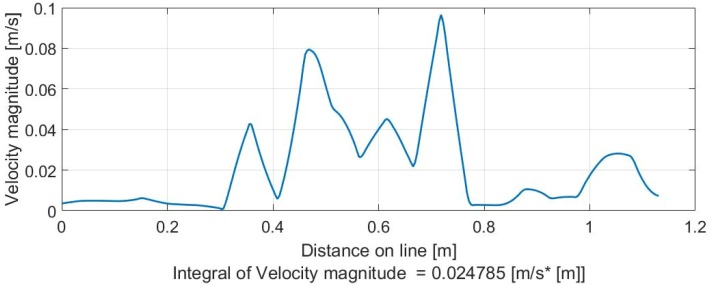
Input (bottom) and output (down) velocity profiles for photo 1b (Figure 14).

**Figure 16:**
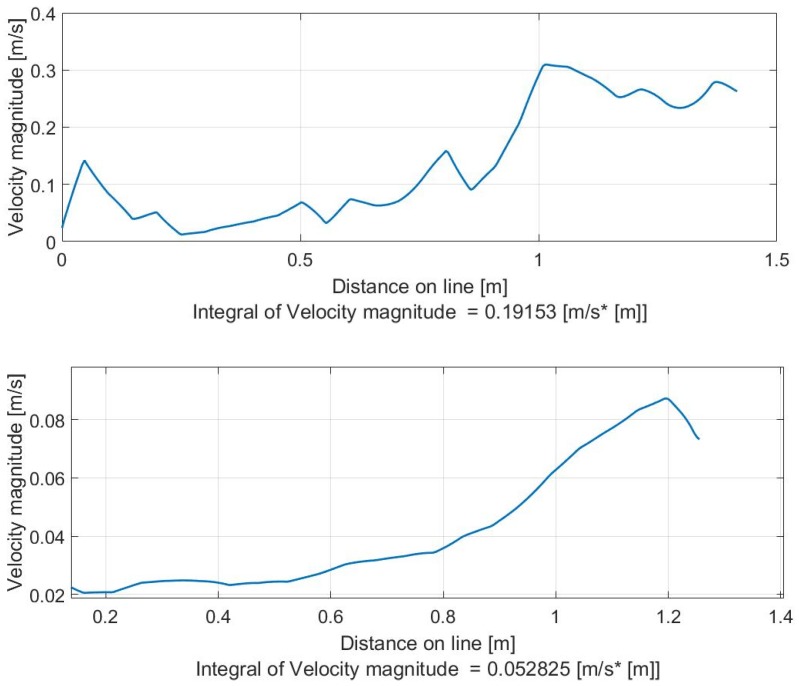
Input (bottom) and output (down) velocity profiles for photo 2b (Figure 14).

The maps of the velocity field can be translated into velocity profiles that show the velocity magnitudes to be considered, given a certain line, as a vision between inlet flow and outlet flow. Figure 15 and Figure 16 clearly explain the results of Figure 14.

To enhance thestatistics, as for instance, September is generally a month with an increasing rainfall, we have applied the algorithm in the same day, 13 September 2016, but with a temporal difference of around 29 min, as shown in Figure 5.

**Figure 17:**
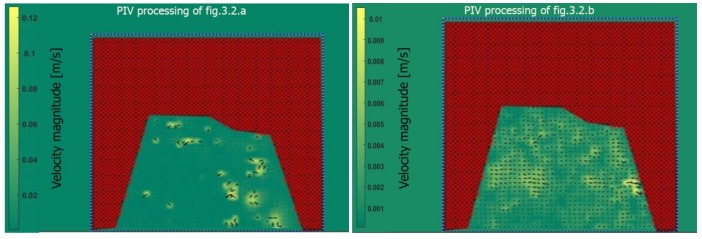
PIV processing of Figure 5 expressing the velocity field.

As we can notice, the algorithm demonstrates clear differences in terms of the velocity field due to different water presence in the two different instants (Figure 17).

### 5.2. Nonparametric Parametric Test Results

The test statistic is the count of the number of observations above the hypothesized median, which therefore has a binomial distribution under the null hypothesis. This enables this method to be invariant against monotone transformations of the data. The sign test applied to the median was performed for unpaired data, for each variable (maximum, minimum, and maximum level) in two periods. The results are reported in Table 4.

Table 5 summarizes the results of the Wilcoxon signedrank test which were applied to examine water level differences temporally.

## 6. Conclusions

This paper has presented an enhanced version of PIV with a masking of image processing that allows the measurement of the velocity field and profiles without geometrical transformation. Geometrical transformation or orthorectification is necessary when imagery is not located in a plane parallel to that of camera surface. As we can notice, from the images included, there is a small distortion that may be corrected since the projection of the river is a little bit distorted due to the oblique viewing angle of the camera. There is no need to proceed with a projective transformation [33] insofar as the processing has compensated for this possible fault. The velocity field is almost in accordance with “ground truth” recovered from the measurements performed in the channel. In general, the difference (deviation) is around of 0.2% which is acceptable due to the low magnitude of the velocity. This is possible because, from the processing, for example of Figure 17, we obtain the following features:

- Mean displacement in *x* and *y* (pixels) = 1.2599, 1.4888
- Maximum displacement in *x* and *y* (pixels) = 4.7382, 10.3942
- Number of valid vectors versus total vectors = 742,1218
- Elapsed time (seconds) = 6.7296875

These features are very interesting, and can allow us to consider the images captured by the webcam and processed by the proposed and enhanced PIV technique, as viable elements to detect the velocity field. However the proposed approach, based on PIV, is not, at the moment, able to exhibit best results in the presence of a major thickness of trash materials; the limitation is due to the performance of the webcam. But in the presence of sophisticated cameras, one mounted as it is now, and another in a tilted position to detect appropriate thickness, the approach can deliver optimized results.

## Figures and Tables

**Table 1:** Selected period data treatment during summer intervals.

N. Range	Period	Image Date	Data Missing
1	06/16/16–07/15/16	1 and 12 July	8 (0.28%)
2	08/02/16–08/31/16	18 August	5 (0.17%)

**Table 2:** River levels in two different ranges within the period of interest.

		Med	Max	Min	St.Dev	Median
range n. 1	Level med [cm]	152.32	158.07	146.97	2.18	152.12
	Level max [cm]	152.80	164.79	147.57	2.15	152.59
	Level min [cm]	151.81	157.81	145.96	2.32	151.68
	Prec [mm]	0.003	1.2	0	0.047	0
range n. 2	Level med [cm]	147.60	156.1	137.88	3.61	147.89
	Level max [cm]	148.15	164.66	138.71	3.58	148.535
	Level min [cm]	147.09	155.88	137.03	3.75	147.31
	Prec [mm]	0.004	2.8	0	0.093	0

**Table 3:** Considered pairs for the Wilcoxon signed rank test.

Pair	Variables
**A**	Level max_1 & Level max_2
**B**	Level med_1 & Level med_2
**C**	Level min_1 & Level min_2

**Table 4:** Sign test results.

Parameter	Range N. 1			Range N. 2		
	Level Med	Level Max	Level Min	Level Med	Level Max	Level Min
Median	152.12	152.59	151.68	147.89	148.53	147.31
N+	1436	1439	1437	1439	1440	1439
N−	1439	1429	1437	1438	1440	1438
Smaller value	1436	1429	1437	1438	1440	1438
*p*-value	0.448	0.348	0.463	0.478	0.507	0.478

**Table 5:** Wilcoxon signed rank test results.

Pair	N+	Expected Value	Variance (N+)	*p*-Value	a
**A**	2621	1438.00	719.00	<0.0001	0.05
**B**	2622	1438.50	719.25	<0.0001	0.05
**C**	2593	1439.000	719.500	<0.0001	0.05
